# Energy-Consumption-Based Life Cycle Assessment of Additive-Manufactured Product with Different Types of Materials

**DOI:** 10.3390/polym15061466

**Published:** 2023-03-15

**Authors:** Osman Ulkir

**Affiliations:** Department of Electric and Energy, Mus Alparslan University, 49250 Mus, Turkey; o.ulkir@alparslan.edu.tr

**Keywords:** additive manufacturing, life cycle assessment, FDM, SLA, energy consumption

## Abstract

Additive manufacturing (AM) or 3D printing technology is one of the preferred methods to ensure sustainability in fabrication. In addition to providing continuity in sustainability, fabrication, and diversity, it aims to improve people’s quality of life, develop the economy, and protect the environment and resources for future generations. In this study, the life cycle assessment (LCA) method was used to determine whether a product fabricated by the AM provides tangible benefits compared to traditional fabrication methodologies. LCA is an evaluation method that provides information on resource efficiency and waste generation, where the environmental impacts of a process can be calculated, measured, and reported throughout the entire life cycle, starting from the acquisition of raw materials, processing, fabrication, use, end of life, and disposal, according to ISO 14040/44 standards. This study examines the environmental impacts of the three most preferred filaments and resin materials in the AM for a 3D-printed product from the start, which consists of three stages. These stages are raw material extraction, manufacturing, and recycling. Filament material types are Acrylonitrile Butadiene Styrene (ABS), Polylactic Acid (PLA), Polyethylene Terephthalate (PETG), and Ultraviolet (UV) Resin. The fabrication process was carried out with Fused Deposition Modeling (FDM) and Stereolithography (SLA) techniques using a 3D printer. Environmental impacts for all identified steps were estimated over the life cycle using the energy consumption model. As a result of the LCA, it was seen that UV Resin was the most environmentally friendly material in the mid-point and end-point indicators. It has been determined that the ABS material also exhibits bad results on many indicators and is the least environmentally friendly. The results support those working with AM in comparing different materials’ environmental impacts and choosing an environmentally friendly material.

## 1. Introduction

The Sustainable Development Goals (SDGs) are a universal call to action by United Nations member states that include goals to be achieved by the end of 2030 [1]. This call aims to build resilient infrastructure, promote sustainable industrialization, and foster innovation. Sustainable fabrication means that businesses carry out their current activities with less harm to the environment and do not endanger their future fabrication [2,3]. In this method, the main objective is to manufacture the product that has the most negligible environmental impact through the optimal use of energy and resources [4].

Life cycle assessment (LCA) is a study in which the environmental impacts and performance of products and services are evaluated throughout their entire lifecycle [5]. In other words, it is a technique used to assess the environmental aspects of a product throughout its lifecycle. The life cycle here covers fabrication, shipment, use, and post-use waste disposal, starting from the supply of raw materials. LCA is a valuable method to ensure a new technology’s viability and applicability and assess whether it provides tangible benefits compared to traditional technologies [6]. Environmental impact can be expressed in terms of global warming or climate change. In addition, LCA results are measurable and allow the comparison of methods. The most basic way to apply the LCA method to additive manufacturing is to produce a 3D model of a part and collect lifecycle data through experiments. These data are then used to calculate the environmental impact [7,8].

Additive manufacturing (AM) or 3D printing is a fabrication technology that allows flexibility in the design and fabrication of parts consisting of multiple materials [9]. AM technology applications have grown in popularity in recent years due to advancements in materials and fabrication methods. This technology is primarily used to fabricate parts for critical industrial areas such as aerospace, automotive, robotics, automation, medicine, biomedicine, the defense industry, and energy [10,11]. It has brought digital flexibility and efficiency to fabrication processes. AM uses data from computer-aided design (CAD) software or 3D object scanners to direct the hardware to deposit the material onto the layer in precise geometric shapes [12]. AM adds materials to create an object, and the geometry is formulated. Unlike traditional fabrication techniques such as milling, turning, machining, play, and shaping, the AM method works with the principle of adding material [13,14].

Researchers and industries use many AM methods in the literature [15,16,17]. Some of these include fused deposition modeling (FDM), powder bed fusion, stereolithography (SLA), digital light processing (DLP), direct metal laser sintering, selective laser sintering, selective heat sintering, electron beam melting, and direct metal laser melting [18,19,20,21]. The FDM method, also known as fused filament fabrication or free-form filament fabrication (FFF), is used in 3D printing technologies to create robust, durable, and dimensionally stable parts with accuracy and repeatability [22,23]. SLA is 3D printer technology that hardens resin using a laser or projection light [24]. In this study, the FDM method was preferred for fabrication with thermoplastic materials, while the SLA method was used for fabrication with UV resin materials.

Nowadays, AM is increasingly used in mass fabrication [25,26]. It offers original equipment manufacturers in various parts of the industry the opportunity to create a distinctive profile based on new customer benefits, cost-effective potential, and the ability to meet sustainability goals [27,28]. This process has many benefits, such as a simple supply chain, longevity of products, and a simple assembly chain. Since AM is a popular fabrication method that is always preferred, its effects on the environment should be minimal [29,30]. For this reason, it is necessary to include sustainability in the process. In terms of sustainability, AM is better than traditional methods. If the methodological infrastructures are not worked on, there will be severe losses in terms of sustainability [31]. We should use technologies such as AM to benefit the world.

Although 3D printing helps to prevent over-fabrication, it remains a problem due to people’s need for more awareness of fabrication and consumption rates. Many people make prototypes that fail in terms of printing standards [32,33]. The process eventually results in more significant amounts of waste. Thus, after some time, running the process takes much time and becomes an efficiency issue in the framework of the circular economy [34]. This process wastes time and requires calculating other factors, such as the transport of residual waste and how much energy it consumes. There has yet to be a study on the LCA of commonly used AM filament materials and UV resins [35,36]. In this study, an LCA of the material and energy consumption of 3D-printed products will be made to solve these problems. Thus, the environmental contributions of the materials will be observed. Another innovation is that the environmental impact of the UVR material will be examined.

Research on the environmental impact caused by the energy consumption of AM primarily focuses on the energy consumption and environmental issues of AM from the life cycle perspective. Although AM is environmentally conscious, there are ways to reduce its environmental impact further. In the manufacturing field, AM is a new fabrication technology that can process complex parts and has high material utilization, which also has the problem of excessive energy consumption and has raised concerns. To address this issue, multi-step optimization methods that enable the AM process for energy efficiency and material consumption are being developed [37,38]. Researchers have optimized AM process parameters to reduce AM’s energy consumption and environmental impact [39,40]. These methods minimize process objectives such as material waste and energy consumption in both the part and layer areas.

Acrylonitrile Butadiene Styrene (ABS), Polylactic Acid (PLA), Polyethylene Terephthalate (PETG), Thermoplastic Polyurethane (TPU), PolyVinyl Alcohol (PVA), and Carbon Fiber are examples of 3D printing materials [41,42]. These materials are thermoplastic-based and are used in FDM technology [43]. In addition to these, UV resins, photopolymer-based, medium viscosity, and thermoset materials that can change shape with light are used in DLP and SLA technology. Burning these materials or throwing them into landfills will negatively affect the environment. The processing of materials before they are reused also requires energy. This contributes to the environmental impact of the materials involved. Therefore, material selection plays an essential role in the circular economy of the materials used in AM.

In this study, an LCA of four popular 3D manufacturing materials, ABS, PLA, PETG, and UV Resin, was conducted from the beginning of the recycling process of a sample product fabricated by the AM method. The purpose of this assessment is to provide decision support for filament material selection based on detailed quantitative environmental impact compared with the LCA of the 3D-printed product. FDM and SLA-based printers were used in the AM. In modeling the stages of the life cycle, an evaluation will be made of both the material consumption and energy consumption of products produced with a 3D printer. Umberto NXT software was used for the modeling process. The obtained results will support those working with AM to compare the environmental impacts of different materials and choose the most environmentally friendly material.

The rest of the paper is organized as follows. In the Materials and Methods section, the equipment used in the AM process, the identification of LCA, and the energy consumption model in this process are explained. In the Results and Discussion section, the results of experimental studies were given. Finally, in the Conclusions section, the article is summarized.

## 2. Materials and Methods

### 2.1. Identifying Life Cycle Stages of Additive Manufacturing

This section describes the determination of the life cycle stages of a material used in the AM process. This process includes how the material is created and operated and how its ecological life ends. The impact of each lifecycle stage is determined by exposure pathways and informed by stakeholder requirements. Given that any material assessment determines the effects of a material hazard, it should be considered in the environment where the printing equipment and resulting products will be used.

In the AM, the life cycle begins with the extraction of raw materials from biological or petrochemical sources, which are processed by chemical conversion and converted into a feedstock for the 3D printer. The person using the 3D printer uses this feedstock to produce the models he designs. The piece whose printing process is completed is then used for the desired application. Depending on the industrial or personal application, the 3D printed part may only sometimes be handled by humans. As a result, the final produced object must be discarded or sent to the recycling process, along with the excess materials from the printing process.

**Figure 1:**
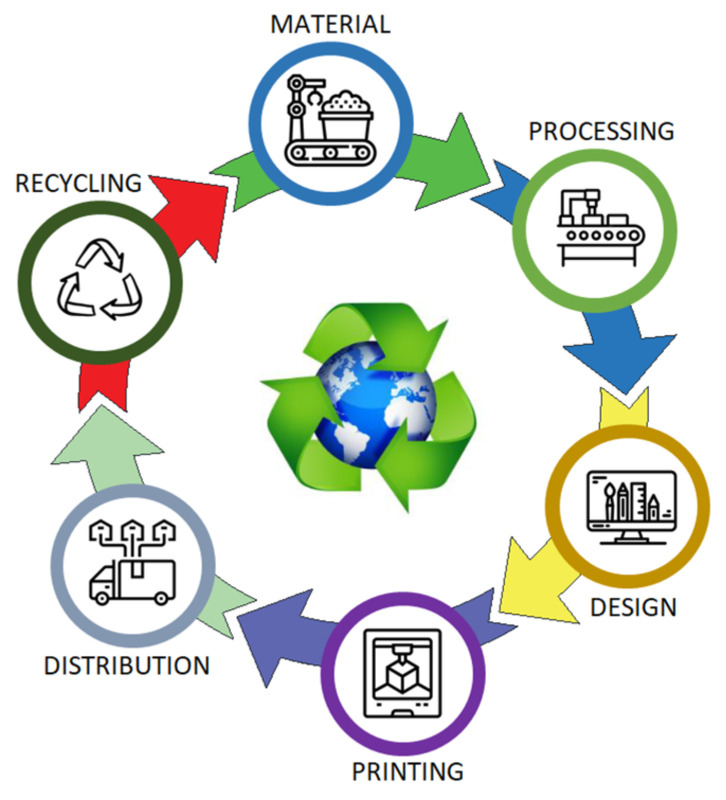
Life cycle of the additive manufacturing process.

The material assessment considers a life cycle assessment (LCA) process that covers energy and emission impacts at all stages. The AM life cycle is shown in Figure 1. From this figure, it is understood that the product fabricated in AM is entirely recyclable. However, it is impossible to recycle a product made entirely with AM. The types of plastic materials used in AM are classified as thermoset plastics, thermoplastics, and elastomers. Thermoset plastics require a curing process where the polymers crosslink each other and form an irreversible chemical bond. As a result of the crosslinking process, polymers cannot be re-melted or remolded. As a result, thermoset plastics cannot be fully recycled as they cannot be re-melted. Resin-based 3D printers, such as the SLA process, use thermoset plastics. Thermoplastics do not go through the curing process during use. This means polymers can be re-melted and recycled. Thermoplastics are widely used in FDM-type 3D printers. As a result, in theory, most prints can be recycled, but in production, this is only sometimes the case. The recycling rate of filament-type materials is much higher than that of UV resin materials. Some recyclable filament thermoplastics are PLA, ABS, PETG, Nylon, and Polycarbonate. Finally, elastomers can be natural, such as plastics and rubber, or synthetic, such as thermoplastic elastomers (TPE). There are also thermoplastic rubbers, a type of copolymer made of rubber and plastic. They are not commonly used in 3D printing and cannot be recycled.

As seen in Figure 1, the life cycle process in AM consists of six steps [44]. The first two steps show the raw material extraction and the processing process that enables this material to be converted into a usable product. AM raw materials are extracted from the soil, derived from bio-based sources, or reused from existing materials. Recycled material sources are more attractive due to their commitment to green design principles. This reveals that some bio-based polymers can have a more significant environmental impact. The model’s design process, which is planned to be produced, is carried out in the third step. The aim is to design the product in 3D and convert it into a 3D printable form. The fourth stage involves the fabrication of the designed product. The fabrication process is completed by selecting the appropriate 3D printer according to the articulated manufacturing method. The product meeting with the user takes place in the 5th stage. This is the last step, where the products are ready to be transported. It includes all the manufacturing and transportation activities required in the product’s filling, packaging, and distribution. Products are either transported to retail outlets or directly to the consumer. Environmental impacts from transport, such as trucks or ships, can also be considered in this step. The final stage is the recycling process. This process includes the energy requirements and the wastes and emissions produced after the product or material has been decommissioned. In this stage, wastes that can be re-evaluated are re-included in the fabrication process through various methods. In other words, it is the recycling of materials that turn into waste after being used in the manufacturing processes as raw materials after different physical and chemical processes are applied.

### 2.2. Life Cycle Assessment

This study used the LCA to investigate the environmental effects of 3D-printed products fabricated from different materials in the AM. It is a tool used to identify, and manage environmental impacts at different life cycle stages, starting with the acquisition of raw materials used in the fabrication of a product or service, including all relevant fabrication and shipment [45]. The ISO 14,040 standard was used as a reference in the LCA. The ISO 14,040 corporate LCA is a comprehensive analysis of the life cycle of some products with sustainability and environmental impacts. It is also a principle that will enable various environmental interactions to encompass countless activities throughout the process.

Umberto software was used to model the AM life cycle stages and then examine and calculate the effects at each stage. This software is one of the leading LCA software solutions for research and education. This research measured and analyzed professionals and is recommended by the industry to determine and the impact of energy and material consumption in the AM. These parameters were taken as a result of a real-time experimental study at all stages of the LCA.

### 2.3. 3D Printing Process

Fused deposition modeling (FDM) and Stereolithography (SLA) were used to fabricate the proposed model. FDM printers produce items by pushing a filament material and flowing it from the nozzle, kept at a specific temperature, by a method such as injection logic. It is possible to create products in different geometries with this method, which is made from thermoplastic materials. The methods used in the AM process are presented in this section.

**Figure 2:**
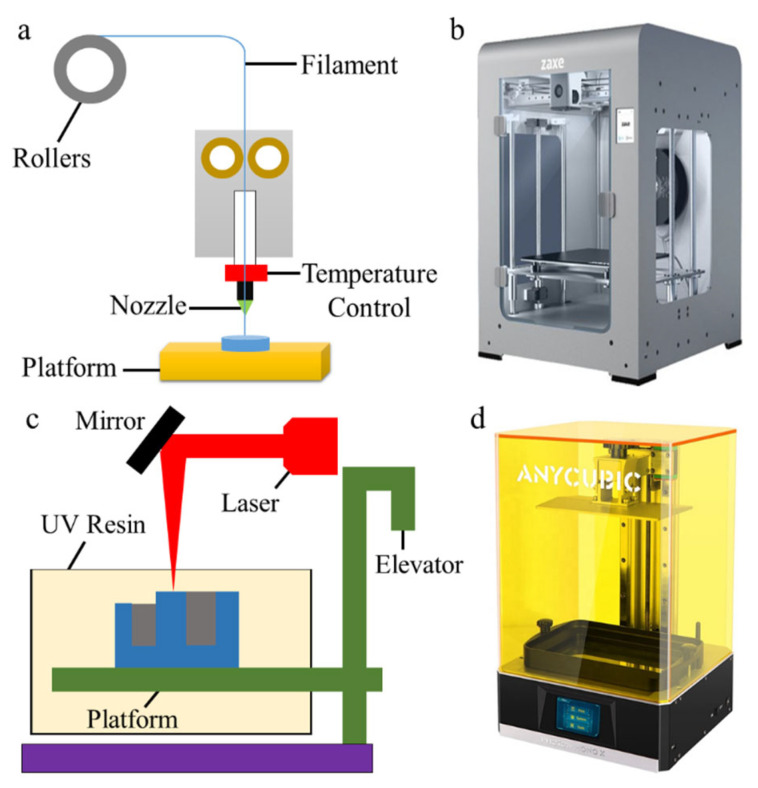
Schematic of the FDM and SLA method: (**a**) FDM fabrication process image; (**b**) FDM-based 3D printer; (**c**) SLA fabrication process image; (**d**) SLA-based 3D printer.

The working principle of FDM technology is shown in Figure 2a. As shown in the figure, the thermoplastic material of a certain thickness, which is wound on a particular reel, is moved with the help of pushers and passes through the nozzle with a temperature control unit at a specific temperature or above. The desired part is produced with the control system. Hundreds of polymer materials, such as ABS, polycarbonate, PETG, and PLA, are used in FDM construction and support materials. This study used a Zaxe Z1 Plus 3D printer (Zaxe, İstanbul, Turkey) with a print volume of 300 × 300 × 300 mm and a layer resolution of 50–400 microns for FDM-based fabrication (Figure 2b). This printer can produce materials such as ABS, PLA, nylon, flex, PETG, carbon fiber, and wood.

SLA is a 3D printing method from the boat photopolymerization family. The SLA technique is based on curing (solidifying) certain regions of the photopolymer resin layer, which is liquid at room temperature, using a point ultraviolet laser beam. The scanning system, which moves under computer control, creates the first layer by traversing (scanning) the laser beam on the resin layer according to the part’s geometry. After one layer is finished, the platform (elevator) on which the part is located is lowered to the thickness of the layer. A new layer of the liquid photopolymer is plastered over the first one with the help of a wing. The curing process continues in order, and the part is produced. After the layers are complete, the piece is removed from the resin pool. While the part is being formed, the structure that serves as support is mechanically separated from the part. The working principle of the SLA system is given in Figure 2c. This study used an Anycubic Photon Mono X 3D printer equipped with a 405 nm laser, 30 μm XY resolution, and 10 μm dynamic Z resolution for SLA-based fabrication (Figure 2d).

**Figure 3:**
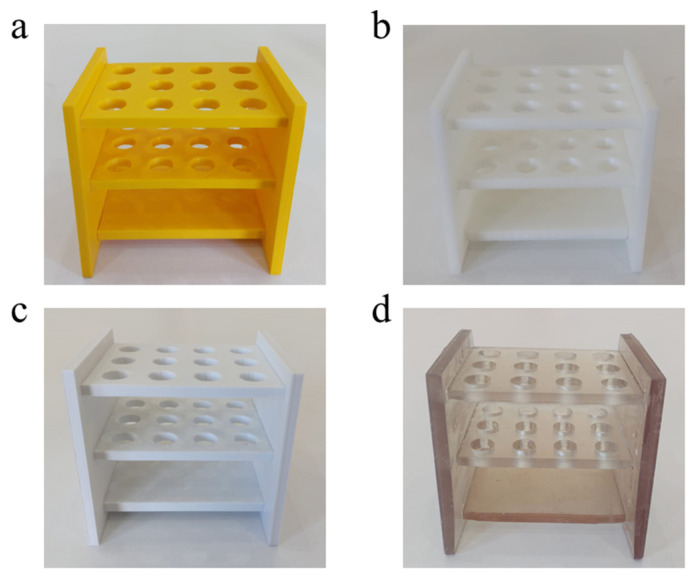
Plastic test tube rack fabricated by AM (**a**) Rack fabricated using ABS (**b**) Rack fabricated using PLA (**c**) Rack fabricated using PETG (**d**) Rack fabricated using UV Resin.

This study aims to analyze, evaluate, and compare the environmental effects of ABS, PLA, PETG, and UV Resin for products produced with AM, which consists of raw material extraction, fabrication, use, and recycling stages, within the framework of their life cycle. The analysis will examine the environmental impacts of the consumption of materials and electricity throughout the life cycle. The functional unit, which will be evaluated within the life cycle framework, has been determined as a plastic test tube shelf consisting of three frames and two borders. These racks are commonly used in laboratories to keep test tubes upright, so equipment is not lost, spilled, or accidentally cracked. The functional units produced with a 3D printer using four materials are given in Figure 3.

First, a 3D solid model of the product is created in the computer environment for the fabrication phase. The completed computer-aided design (CAD) file is saved in Standard Triangle Language (STL) format. This file is then transferred to the slicer software. In this study, XDesktop (1.9.28) and Anycubic Photon Workshop software (version 1) were used for slicing with FDM and SLA methods, respectively. In this software, the printing parameters given in Table 1 are defined, and the slicing process is completed. The amount of material the model will consume and the fabrication time are shown in Table 1. The fabrication file is created using the GCODE file obtained at the end of the slice. This file translates the model into a language the 3D printer can understand. Finally, the calibration processes for the printer are completed, and the 3D printer is ready for fabrication. Using FDM and SLA-based 3D printers, this process is repeated for each part from start to finish. A single-phase energy meter was used to measure energy consumption during fabrication. At the end of each fabrication, these values were recorded, and the total energy consumption was calculated as kWh.

**Figure 4:**
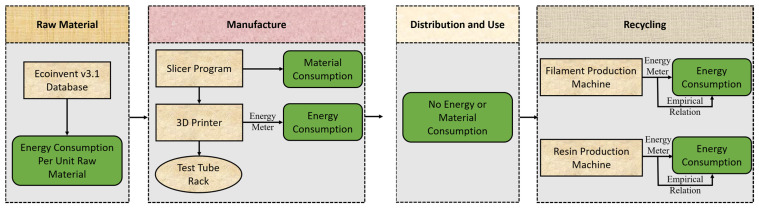
Flow chart showing the life cycle energy analysis model for FDM and SLA additive manufacturing methods.

The flow chart showing the life cycle energy analysis model for FDM and SLA additive manufacturing methods is shown in Figure 4. The raw material process covers the processes required to remove ABS, PLA, PETG, and UV Resin materials from the environment and is ready for use in the 3D printer. The fabrication process includes the stages of 3D printing. The energy consumed by the printer and the amount of material used is considered. Since the product is passive, there was no energy consumption during the usage phase. In addition, the transportation processes of raw materials and 3D-printed products are not considered. As the last step, recycling includes converting the products back into filament and resin form. In this step, the process of converting the product fabricated and used by the consumer into a reusable material form is carried out. FDM-based materials are produced with plastic extrusion machines. Extrusion systems work with the logic of transferring the plastic granule particles placed in a funnel to the heater nozzle side at the end of the funnel with the help of a screw shaft and melting it by passing through heat treatment [46]. On the other hand, SLA-based material is produced by depolymerizing the produced parts and dissolving them in a recycling solvent [47]. Many chemical processes and heating devices are used here. In the current study, the recycling process is modeled using empirical relationships instead of real-time experimental studies. 

### 2.4. Energy Consumption Modelling

The data required for each stage of the life cycle assessment process were measured in real time as a result of experimental studies and taken from the life cycle database. The amount of material and energy consumption data used for ABS, PLA, PETG, and UV resin materials in the fabrication phase of 3D printing with FDM and SLA methods are given in Table 2. The data for the raw material extraction stage were obtained from the Umberto Ecoinvent v3.1 database. In contrast, the data for the fabrication stage was acquired by 3D printing the plastic test tube rack. While the energy consumption data were collected with a single-phase energy meter, the material consumption amounts were taken from the slicing program of 3D printers.

Due to the passive nature of the developed test tube rack system, we neglected plastic deformation during its life cycle. We assumed all the material could be recycled during the recycling phase. The amount of energy consumed while extruding the filament material during fabrication in the 3D printer is calculated as follows [48]:

(1) $$E=C_{p}\times \Delta T$$

$C_{p}$ represents the material’s specific heat capacity, and ΔT represents the temperature increase. It is seen that the amount of energy consumed is directly proportional to these two variables. The total energy consumed in the recycling phase is calculated as in Equation (2) [49]. The total energy here is the sum of the energy spent to heat the 3D printer first and the energy consumed to extrude the filament. The initial heating energy is independent of the material and has been measured experimentally. These values are different for FDM and SLA methods. The FDM is 0.07 kWh for ABS, PLA, and PETG. It is calculated as 0.03 kWh for SLA.

(2) $$E_{\mathrm{LC}}=E_{\mathrm{initial}\_\mathrm{heating}}+E_{\mathrm{extrusion}}$$

$E_{\mathrm{LC}}$, $E_{\mathrm{initial}\_\mathrm{heating}}$, and $E_{\mathrm{extrusion}}$ represent the recycling energy, the energy consumed during the preparation of the 3D printer for the printing process, and the energy spent for extrusion, respectively. The amount of energy consumed during extrusion was calculated by equating the proportionality constant for different materials, regarding the polypropylene data. It should be noted that the calculated energy is the constant volume of filament extruded each time. For this reason, the amount of energy required to extrude a unit gram of PLA material is calculated with the following equation [48]:

(3) $${\left(\frac{E}{C_{p}\Delta T}\right)}_{\mathrm{PP}}={\left(\frac{E}{C_{p}\Delta T}\right)}_{\mathrm{PLA}}\;{\left(\frac{0.1}{1920\times 175}\right)}_{\mathrm{PP}}={\left(\frac{E}{1800\times 165}\right)}_{\mathrm{PLA}}\;E_{\mathrm{PLA}\;\mathrm{for}\;2.73\;g}=0.0883\;\mathrm{kWh}\;E_{\mathrm{PLA}\;\mathrm{per}\;\mathrm{gram}}=\frac{0.0883}{2.73}=0.0323\;\frac{\mathrm{kWh}}{g}$$

The total energy for recycling the functional unit is calculated as in Equation (4) by multiplying the energy needed per gram by the weight of the functional unit and adding the energy required for initial heating. By repeating this process for each material, the values in Table 2 are obtained.

(4) $${\left(E_{\mathrm{LC}}\right)}_{178.59g}=0.07+\left(0.0323\times 178.59\right)=5.83\;\mathrm{kWh}$$

It is possible to examine the environmental effects of any product with numerical data using LCA. The energy and material flow model in Umberto NXT software, which closes the stages shown in Figure 4, was used. There are two approaches to evaluating environmental impact within the context of LCA. These are the mid-point and end-point assessments. The end-point category represents ecological damage due to a stimulating natural environment or human health. The mid-point category aims to cover the environmental issue between inventory (emissions, etc.) and eventual damage to the conservation area. Impact assessment follows a cause–effect chain from stock to at least mid-point indicators, and optionally continues with additional cause–effect modeling to evaluate end-point outcomes. Mid-point and end-point indicators were calculated using the ReCiPe method for life cycle impact assessment. The primary purpose of this method is to convert long-life cycle inventory results into a limited number of indicator scores. These indicator scores define the severity of an environmental impact category.

For three filaments and one resin material, both mid-point and end-point assessments were carried out with selected indicators using measured and calculated data from the raw material extraction, fabrication, and recycling stages. This study’s mid-point assessment covered human toxicity, climate change, fossil depletion, ozone mining, particulate matter formation, freshwater ecosystems, terrestrial acidification, water depletion, photochemical oxidant formation, ionizing radiation, and metal depletion. In contrast, the end-point assessment examined ecosystem quality, human health, and resources.

## 3. Results and Discussion

### 3.1. Experimental Results

**Figure 5:**
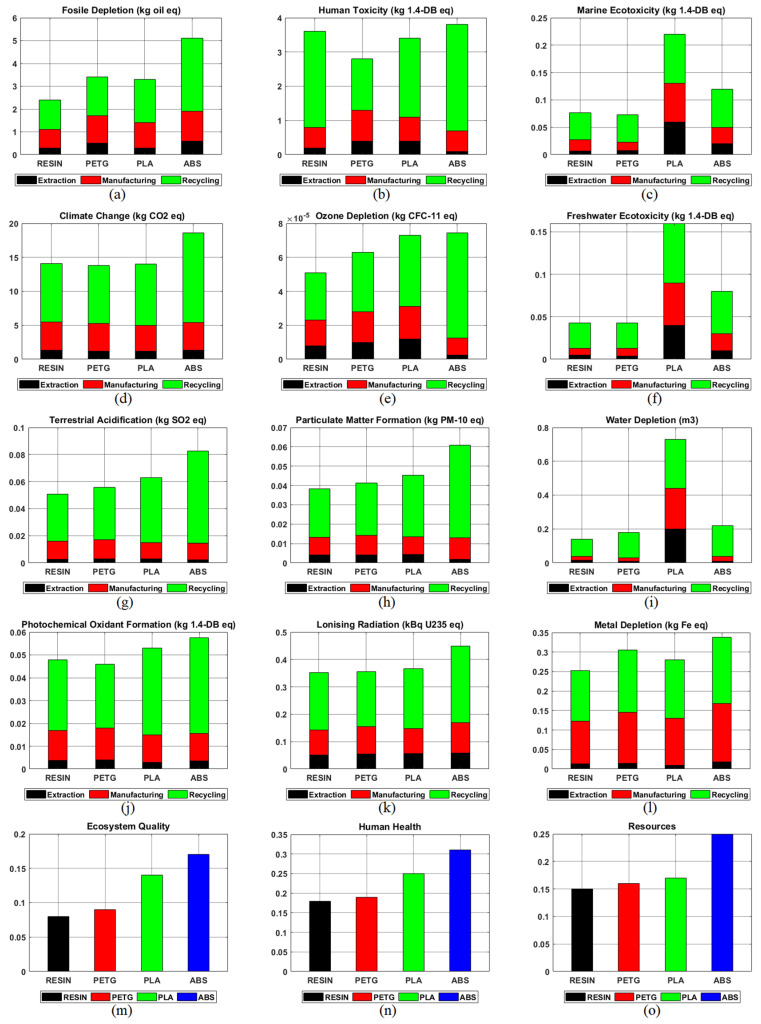
Mid-point and end-point indicator results in the lifecycle assessment process (**a**) Fossil depletion (**b**) Human toxicity (**c**) Marine ecotoxicity (**d**) Climate change (**e**) Ozone depletion (**f**) Freshwater ecotoxicity (**g**) Terrestrial acidification (**h**) Particulate matter formation (**i**) Water depletion (**j**) Photochemical oxidant formation (**k**) Ionizing radiation (**l**) Metal depletion (**m**) Ecosystem quality (**n**) Human health (**o**) Resources.

As a result of the analyses performed for the life-cycle evaluation of AM technology, the results for each of the four materials are given in Figure 5. These materials are Resin, PETG, PLA, and ABS, which are the most preferred among FDM and SLA technologies. The contributions of LCA’s raw material extraction, manufacturing, and recycling stages on materials were examined. Twelve indicators were referenced in the mid-point assessment, while three indicators were used in the end-point assessment. As seen in Figure 5, these indicators are fossil depletion, human climate, marine ecotoxicity, climate change, ozone depletion, freshwater ecotoxicity, terrestrial acidification, particulate matter formation, water depletion, photochemical oxidant formation, ionizing radiation, metal depletion, ecosystem quality, human health, and resources.

As a result of the analyses conducted with PLA material, it was determined that the recycling phase had the highest impact on six of the twelve indicators in the mid-point evaluation process. In other words, the recycling effect was over 50%. The data in which the raw material extraction stage of the PLA material is the highest can be seen in Figure 5c,f,i. It has been determined that the raw material extraction step has the highest contribution to marine ecotoxicity, freshwater ecotoxicity, and water depletion. It is seen that the raw material extraction stage in water-related parameters is relatively high in PLA compared with other parameters. This high rate may be related to the extraction, as PLA is produced from raw materials such as cornstarch and sugarcane. As this impacts water depletion and the freshwater system, it can harm freshwater species.

The mid-point evaluations made for ABS material determined that the recycling stage had the highest effect on all indicators. This is because ABS material consumes more energy in the recycling phase than other material types. It was determined that the raw material extraction stage had the lowest effect on all indicators except marine and freshwater ecotoxicity. The fabrication phase consistently affects all indicators with an average range of values (15–20%).

PETG material had similar results to ABS. As a result of the mid-point evaluation of this material, it is seen that the recycling stage has the highest environmental impact on all indicators. Moreover, the raw material stage has the lowest impact of all indicators. Finally, the production process is seen to have a consistent effect of approximately 25% on all indicators.

The UV resin material exhibited similar results to PETG. It has been determined that the recycling stage of this material has the highest environmental impact. In particular, the recycling process had a high human toxicity indicator. This may be due to the high amount of toxic material emitted by the material during production. As with PETG, the raw material extraction process has the lowest environmental impact. In the production phase, a consistent effect of 20% was observed on average, but a low environmental impact was observed in water-related parameters.

The end-point assessment results for Resin, PETG, PLA, and ABS materials are shown in Figure 5m–o. In this evaluation, indicators of ecosystem quality, human health, and resources were chosen. Although similar environmental effects were observed in all indicators, the impact of ABS material is relatively high. In contrast, the resin material is at a low level.

Among the materials, ABS has a high environmental impact in all indicators in the production and recycling stages thanks to its material properties. Made of petroleum-based plastic material, ABS has good resistance to heat and chemicals and is easy to process, thanks to its strong and durable structure, even at low temperatures. Although the effects of UV resin and PETG materials are close, a low environmental impact has been observed. The effect of the resin in terms of human health and resource indicators is lower. Furthermore, PLA ranks lower than ABS in the ecosystem quality indicator and has a high negative environmental impact.

### 3.2. Discussion

Nowadays, the 3D printing technology process is drastically changing the way of performing production work and the way people live. Conducting energy and related environmental impact analyses on this critical technology is mandatory and essential. In this context, energy modeling for FDM and SLA, the most common AM processes, was first examined from a life cycle perspective, and necessary analyses were carried out. As a result, findings that can support material selection in 3D manufacturing have been determined. In the literature, the environmental impact of parts produced using 3D printing, especially using FDM-based materials, has been investigated through life cycle analysis [50,51]. However, there is a severe deficiency, especially in UV resin material. In the current study, in addition to ABS, PLA, and PETG materials, the production included UV Resin material, and its environmental effects were examined. In Section 3.1, the effects of materials on the environment are presented in different aspects. The results obtained are similar to those reported in the literature [52,53].

Technological developments will enable the production of 3D printers that provide faster and more efficient performance. The existing limitations regarding fabrication, support structures, long production time, and delamination caused by temperature and chemical fluctuations will be improved with FDM and SLA. Many data are needed to further analyze the impact of different part designs, important and influential parameters, and equipment selection. The energy consumption and cost of 3D machine building, maintenance, and repair can be included in the life cycle analysis. The collaboration of FDM and SLA 3D printers to fabricate a product in an energy-efficient manner can also be envisioned as future work.

## 4. Conclusions

AM is the preferred technology for environmental friendliness and sustainability. The popularity of this technology, which has been developed as an alternative to traditional fabrication methods, is increasing daily. The most critical parameter in this technology is selecting the material to be used because the choice of material is crucial for defining the quality of the manufactured product and the environmental damage it produces. This study investigated the environmental effects of the four most preferred material types in AM using two 3D printers. These materials are ABS, PLA, PETG, and UV Resin. FDM and SLA-based 3D printers were used in the AM process. A life cycle assessment was carried out to determine environmental impacts. The effects of the product during the AM life cycle were analyzed with this process.

The LCA process has been studied in three stages: Raw material extraction, fabrication, and recycling. Environmental impacts for all identified steps have been estimated over the energy consumption model. The data for the raw material extraction stage were obtained from the Umberto Ecoinvent v3.1 database. In contrast, the data for the fabrication stage were acquired by 3D printing a plastic test tube rack. While the energy consumption data were collected with a single-phase energy meter, the material consumption amounts were taken from the slicing program of 3D printers.

As a result of the analysis, it has been seen that UV Resin material is the most environmentally friendly regarding mid-point and end-point indicators. ABS material was also found to have poor results in many indicators and is the least environmentally friendly. It has been determined that PETG material is the most environmentally friendly material after UV resin. PLA material, on the other hand, had a negative effect, especially on water-related parameters. For all materials, the recycling stage in LCA resulted in the highest environmental impact.

When the data obtained from experimental studies and analyses are examined, there is less material and energy consumption in AM made with the SLA technique. Especially in FDM technology, pre-treatments used to reach the high table and nozzle temperatures consume significant energy. In addition, while the fabrication process takes approximately 450 min in SLA, this value is 550 min in FDM. Especially at this point, SLA-based fast printing devices will provide time, cost, and energy advantages. The results obtained from the LCA will support those working in AM in comparing the environmental impact of different materials and choosing an environmentally friendly material.

In the following study, the environmental impact process in AM will be extended using different materials such as carbon fiber and metals. In addition, a comprehensive study of environmental effects can be carried out using other 3D fabrication techniques, such as digital light processing powder bed fusion and selective laser sintering.

## Figures and Tables

**Table 1:** Printing parameters of materials used for fabrication in FDM and SLA 3D printers.

Properties	ABS	PLA	PETG	Resin
Infill Volume (%)	40	40	40	40
Layer Thickness (µm)	300	300	300	30
Nozzle Temperature (°C)	230	210	250	-
Table Temperature (°C)	90	80	100	-
Exposure Time (s)	-	-	-	15
Bottom Exposure Time (s)	-	-	-	105
Bottom Layers	-	-	-	5

**Table 2:** Real-time material and energy consumption data for LCA.

Materials	Material Consumption (g)		Manufacture Energy Consumption (kWh)	Recycling Energy Consumption (kWh)
ABS	91.23	87.36	1.98	6.95
PLA	98.76	94.27	1.54	5.83
PETG	89.15	84.29	1.83	4.48
UV Resin	90.46	78.63	1.13	3.59

## Data Availability

Data is contained within the article.
